# Case Report: Simultaneous *Paragonimus skrjabini* infection in twin girls with spontaneous emergence of a juvenile worm from the eyelid of the elder sister

**DOI:** 10.3389/fped.2025.1708963

**Published:** 2025-12-19

**Authors:** Jintao Liu, Liyun Huang

**Affiliations:** Department of Ophthalmology, Xinqiao Hospital of the Army Medical University, Chongqing, China

**Keywords:** metagenomic next-generation sequencing, *Paragonimus skrjabini*, praziquantel, raw crabs, twin sisters

## Abstract

We reported a rare case of paragonimiasis occurring in twin sisters who had eaten raw crabs 7 months ago. The elder sister complained of eyelid swelling and migratory lumps, while the younger sister was asymptomatic. Laboratory tests showed eosinophilia and elevated levels of inflammatory indicators in the two sisters. The brain MRI of the elder twin showed hyperintensity in the frontal lobe, suggesting cerebral hemorrhage. The chest CT image of the twins showed pulmonary involvement. Enzyme-linked immunosorbent assay (ELISA) for serum antibody test and metagenomic next-generation sequencing (mNGS) of subcutaneous tissue from the eyelid—obtained via sterile puncture aspiration under local anesthesia—confirmed *Paragonimus skrjabini* infection. After praziquantel treatment, both of the sisters recovered. This study aims to enhance clinical awareness and highlight the application of advanced molecular diagnostic technologies for identifying rare parasitic infections.

## Introduction

Paragonimiasis is a neglected food-borne parasitic disease caused by lung flukes. *Paragonimus skrjabini* is one of the major pathogens causing paragonimiasis in China with distinct pathogenic characteristics. Since humans are not the natural definitive hosts, the juvenile worms can persist in the human bodies for an extended period and show strong migratory ability, leading to the occurrence of ectopic localization and various clinical manifestations (1). Due to the atypical migration path, it often causes local or even systemic inflammatory response, mimicking tuberculosis, pneumonia, meningitis, and orbital inflammation. Therefore, *P. skrjabini* infection is easily misdiagnosed in clinical practice.

## Case series

**Figure 1:**
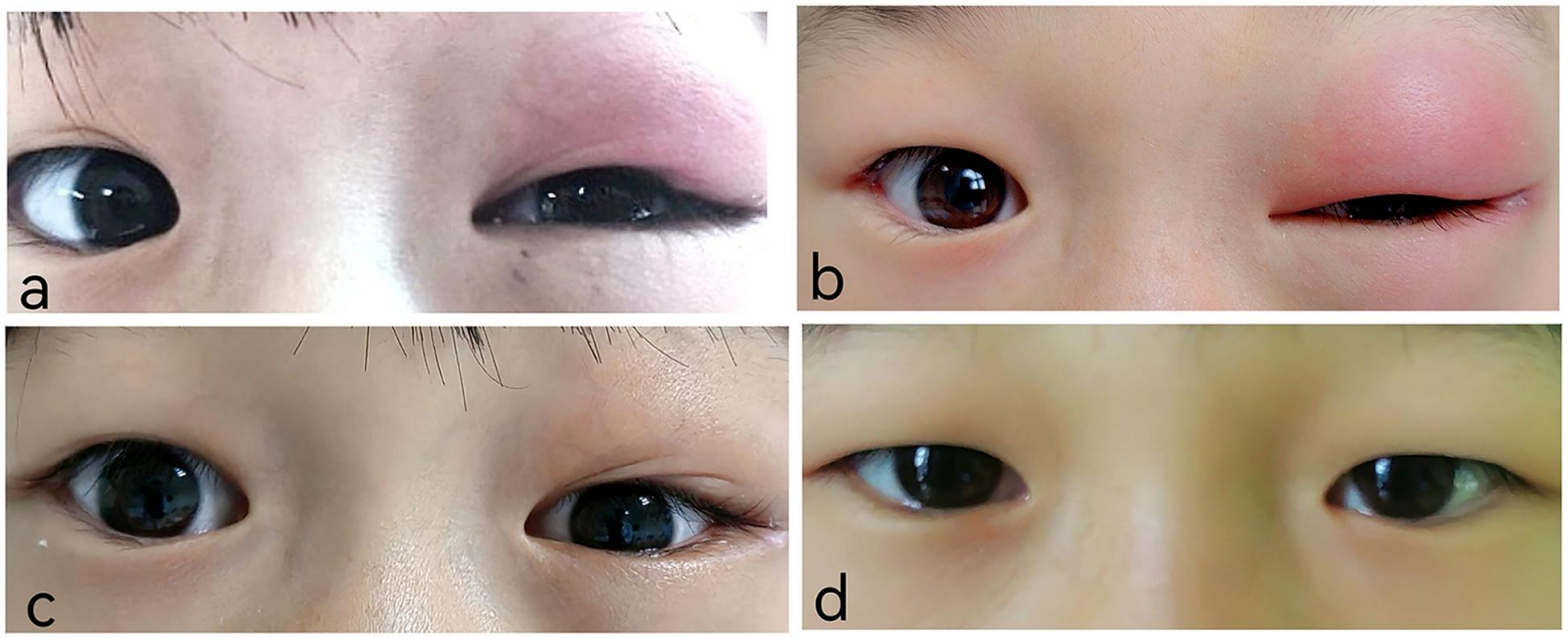
The eyelid appearance of the twin sisters **(a)** the elder twin sister had upper eyelid swelling. **(b)** The elder twin sister had worsening swelling of the upper and lower eyelids. **(c)** Three days after oral administration of praziquantel, the swelling of the elder twin sister's upper and lower eyelids significantly resolved. **(d)** The eyelid appearance of the younger twin sister.

**Figure 2:**
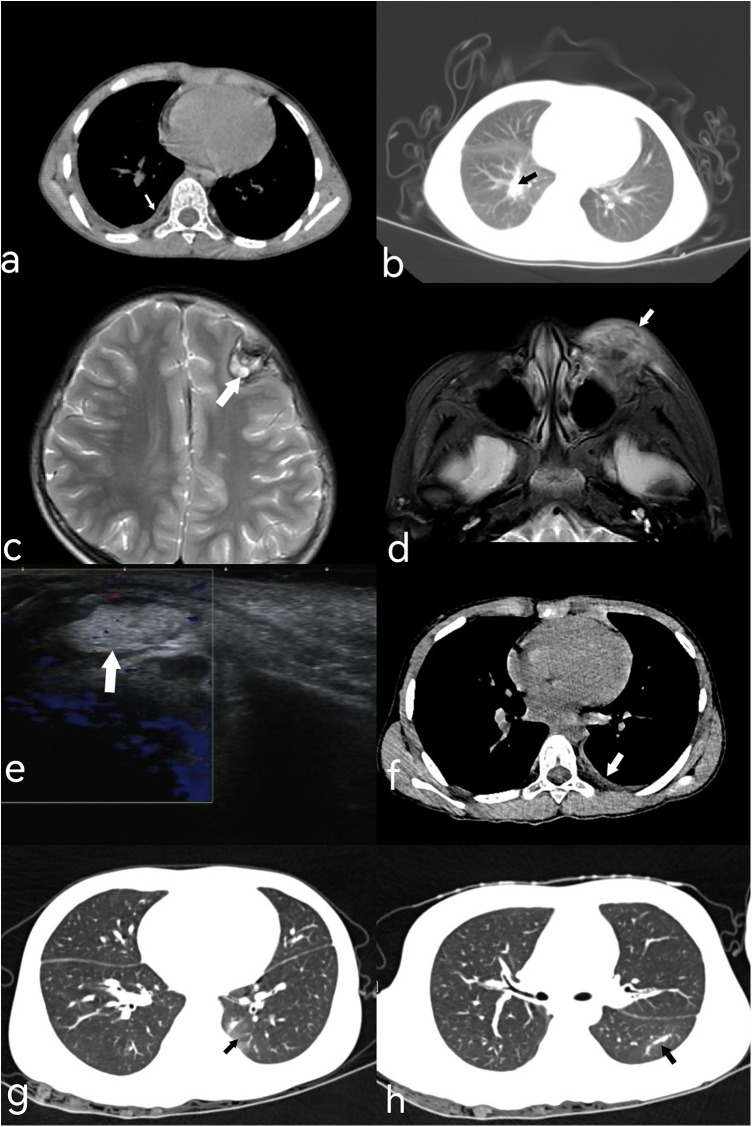
The imaging examination images of the twin sisters. **(a)** Chest CT (elder twin): Small right pleural effusion (white arrow). **(b)** Chest CT (elder twin): Patchy hyperdense opacities in right lower lobe (black arrow). **(c)** Brain MRI (3.0T, T2W-TSE, elder twin): Flaky T2 hyperintensity in left frontal lobe with circumferential marginal hypointensity (white arrow). **(d)** Orbital MRI (3.0T, OCor STIR, elder twin): Left periorbital soft tissue swelling with T2 hyperintensity (white arrow). **(e)** Ocular color Doppler ultrasound (elder twin): Irregular hyperechoic mass under left eyelid (white arrow). **(f)** Low-dose chest CT (younger twin): Slight left pleural thickening and small pleural effusion. **(g,h)** Chest CT (younger twin): Lesions in dorsal/posterior basal segments of left lower lobe (black arrow).

An 8-year-old girl from Chongqing, China, presented with persistent left eyelid swelling for one month. She reported accidental exposure of her left eye to seasoning powder from instant noodles one month earlier. The eye was rinsed with tap water immediately. Two days later, she developed upper eyelid swelling and conjunctival congestion (Figure 1a). Treatment with erythromycin ointment and chloramphenicol eye drops failed to improve her symptoms. She visited the People's Hospital of Fengjie County, where an orbital CT examination was performed, but no treatment was given. Without improvement for 2 weeks, she developed postprandial vomiting and underwent left inguinal hernia repair. During hospitalization, the swelling of her left eyelid shifted from the upper to the lower lid. After-discharge, she visited the Children's Hospital of Chongqing Medical University, where ocular ultrasound revealed a subcutaneous inflammatory lesion in the left lower eyelid with viscous exudate, but no treatment was initiated. Upon presentation to our outpatient clinic, both upper and lower eyelids were markedly swollen (Figure 1b). No significant past medical history was noted. Her physical examination showed a palpable irregular subcutaneous mass on the left upper and lower eyelids which was well marginated, non-tender, with some mild tenderness at the lateral canthus containing a small subcutaneous nodule. Ocular motility was intact. Chest computed tomography (CT) revealed right lower lobe pneumonia with minimal pleural effusion (Figures 2a,b). Abdominal ultrasound study revealed no abnormality. Laboratory tests showed leukocytosis (26.11 × 10^9^/L), marked eosinophilia (71.7%, absolute eosinophil count 16.71 × 10^9^/L), thrombocytosis (465 × 10^9^/L), and elevated erythrocyte sedimentation rate (28 mm/h). Stool examinations for ova and parasites were negative. Brain magnetic resonance imaging (MRI) demonstrated patchy high signal intensity in the left frontal lobe, suggesting hemorrhage (Figure 2c). The differential diagnosis included inflammatory pseudotumor, orbital cellulitis, hordeolum, Kimura disease or parasitic infection. An orbital MRI showed abnormal signals in periorbital and retrobulbar areas that were consistent with an infectious process (Figure 2d). Orbital ultrasonography showed an irregular hyperechoic mass without flow, possibly due to inflammatory exudation (Figure 2e). A sonographic diagnosis of a tumor could be excluded.

**Figure 3:**
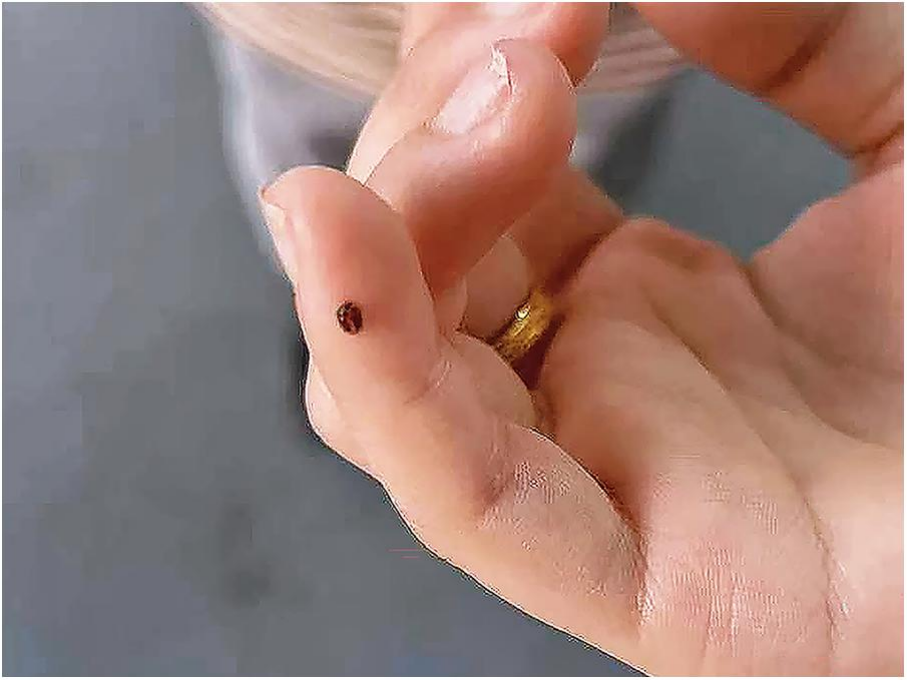
The appearance of the worm that crawled out of the elder twin sister's eyelid. (This image was provided by the patient's family).

**Figure 4:**
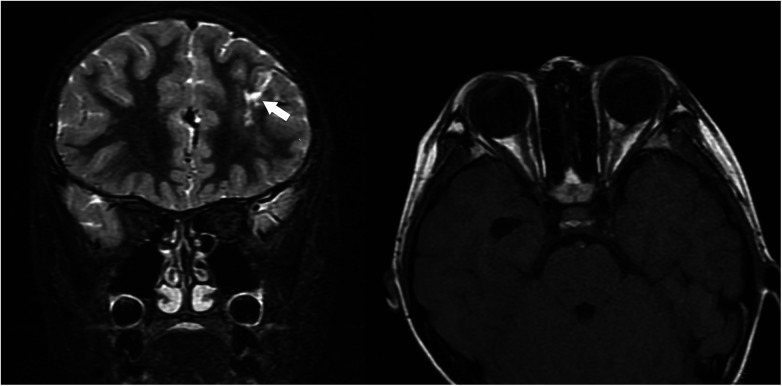
One month after anthelmintic treatment, orbital MRI of the elder twin sister demonstrated marked improvement in left periorbital soft tissue swelling and reduced extent of the left frontal lobe patchy shadow compared with prior imaging. (Left: Coronal T2-weighted orbital MRI, lesion indicated by the white arrow; Right: Axial T1-weighted orbital MRI).

On further questioning, the patient and her twin sister caught crabs from a creek and ate them raw while playing seven months before onset of symptoms, but they did not share the crabs with their family. Since evidence of systemic involvement and CT findings were noted, parasitic infection was considered most likely. mNGS analysis of the eyelid subcutaneous tissue puncture sample yielded 33 sequencing reads matching the genus *Paragonimus* (relative abundance of parasitic sequences: 100%), with 32 specifically annotated to *P. skrjabini*, confirming its infection*.* Notably, 24 h after the eyelid puncture biopsy, an active brown worm the size of a coffee bean emerged from the biopsied trace of her left eyelid (Figure 3), though it was not preserved for identification. ELISA for serum antibody test revealed IgG antibody-positive against *Paragonimus* in both sisters. The younger sister, who was asymptomatic but exposed to the same risk factor (Figure 1d), also showed eosinophilia (43.3%, absolute eosinophil count 5.78 × 10^9^/L), leukocytosis(13.36 × 10^9^/L), thrombocytosis (398 × 10^9^/L), and chest CT abnormalities, including inflammatory lesions in the left lower lobe and left pleural thickening with a small amount of pleural effusion (Figures 2f–h). The brain CT was normal. Multidisciplinary consultation confirmed the diagnosis of paragonimiasis in both twin sisters. Given that the pulmonary imaging exhibited radiological characteristics typical of paragonimiasis, and the diagnosis was validated by a multidisciplinary consultation, antiparasitic treatment was initiated. Both twin sisters received antiparasitic treatment with praziquantel (25 mg/kg orally three times daily for 3 days). The elder sister's eyelid swelling improved markedly after treatment (Figure 1c). One month later, when the elder sister came with her father for a follow-up, her white blood cell count returned to normal (8.36 × 10^9^/L), the eosinophil percentage had decreased from the previous value (21.1%, absolute eosinophil count 1.76 × 10^9^/L), and the platelet count had decreased to 385 × 10^9^/L. Orbital MRI showed that the degree of left periorbital soft tissue swelling had significantly improved compared with the previous examination, and the extent of the patchy shadow in the left frontal lobe had narrowed (Figure 4).

## Discussion

We report two rare clinical features of *P. skrjabini* infection: spontaneous emergence of a worm from the eyelid and marked clinical heterogeneity in monozygotic twin sisters. These findings expand the clinical spectrum of paragonimiases and highlight diagnostic and pathogenic complexities.

*Paragonimus skrjabini* is one of the most important species capable of infecting humans (2). Unlike *P. westermani*, which primarily causes pulmonary disease, *P. skrjabini* typically presents as a pleural or ectopic infection, with pulmonary parenchymal involvement being rare (3). *Paragonimus* metacercariae reside in freshwater crabs. As accidental hosts, humans acquire infection with the metacercariae by consuming raw crabs; subsequently, the metacercariae excyst in the stomach or intestines to form juvenile worms, which can migrate to extrapulmonary sites such as the pleura, subcutaneous tissue, and even the central nervous system (4, 5). This ectopic migration contributes to non-specific clinical manifestations, often mimicking tuberculosis, meningitis, or tumors, and increases the risk of misdiagnosis (6, 7). The clinical features of ocular paragonimiasis shown in 6 reports including 32 cases are summarized in Table 1. All these cases were ocular lesions associated with paragonimiasis, which can be classified into orbital, eyelid, and intraocular types based on the ocular sites invaded by *Paragonimus*. Children and adolescents are the main affected populations, with unilateral proptosis being the most common symptom, some cases are accompanied by eyelid swelling and conjunctival edema; severe cases present with retinal detachment, elevated intraocular pressure (33–50.62 mmHg), and visual impairment (best-corrected visual acuity as low as hand motions). In a few cases, parasites can be detected intraocularly (in the anterior chamber or subconjunctivally) or within the orbit. Elevated white blood cell count and eosinophilia are typical laboratory features; some patients have systemic manifestations such as pulmonary abnormalities (pleural effusion, chronic lung lesions) and abdominal nodules (8–13). The gold standard to diagnose paragonimiasis is the pathological confirmation of *Paragonimus* worms, eggs, or eosinophilic granulomas in sputum, feces, biopsy specimens, or resected tissues (14–16). Since *P. skrjabini* persists as juvenile worms in humans, egg- or adult worm-targeted diagnostic approaches are not fully suitable. Detection of anti-*Paragonimus* antibodies or of circulating *Paragonimus* antigens by ELISA serve as key screening tools, while metagenomic next-generation sequencing (mNGS) enhances the diagnostic yield of ectopic infections (17–19). Praziquantel is the first-line antiparasitic therapy, administered orally at 25 mg/kg three times daily for 3 days (20). Combined surgical intervention is indicated for severe ectopic infections or concurrent complications (15, 16). We highlight the importance of combining information from epidemiological, clinical, and molecular data in the diagnosis of paragonimiasis. Both children ate raw crabs in an endemic region—Fengjie County, Chongqing, but had different disease severity. The elder sister presented with ocular involvement and suspected neurological involvement, while the younger sister remained relatively asymptomatic, except for imaging abnormalities. In this case, the simplest explanation for the difference in the clinical manifestations of the twin sisters is the possible difference in the number of metacercariae they ingested by eating raw crabs. Just by chance, the elder sister might have ingested a larger number of parasites causing multiple lesions in various organs. Possible differences in the immune status of monozygotic twins mediated by epigenetic regulation are a fascinating topic. However, this study lacks relevant experimental data, so this is only a hypothetical speculation that requires verification by further studies.

**Table 1:** Clinical features of ocular paragonimiasis.

Reference	Reported by	Age	Gender	Core ocular signs	Key systemic manifestations
(8)	Zhang et al.	12 y	Male	Unilateral proptosis (18 mm); VA: hand motion; retinal detachment (B-US); conjunctival edema	Eos 27%; right encapsulated pleural effusion
(9)	Wang et al.	13 y	Male	Anterior chamber fluke (2 mm × 3 mm); VA: hand motions; retinal detachment; IOP 43.3 mmHg	WBC17.4 × 10^9^/L; no eggs in sputum/stool
(10)	Cai et al.	10 y	Female	VA 0.5; orbital swelling/proptosis; optic disc edema	WBC9.8–17.0 × 10^9^/L (eos11%–32%); old TB (fluoroscopy)
(11)	Lu et al.	21 y/10 y	Both M	Case1: Proptosis (21 mm), subconjunctival fluke; Case2: Conjunctival edema/fluke	Case1: Eos 20%–32%, old lung lesions; Case2: Abdominal nodules
(12)	Luo et al.	2–47 y	15M/5F	Unilateral involvement (19/20); proptosis (11–30 mm); nodules (3 flukes extracted)	Eos 6%–62%; cough/chest pain
(13)	Jiang et al.	4–11 y	3F/4M	Anterior chamber fluke (3 cases, IOP 33–50.62 mmHg); proptosis (16 mm, 4 cases)	WBC12.0–26.0 × 10^9^/L (eos11%–44%); pleural effusion (Case3)

y, years old; F, female; M, male; VA, visual acuity; WBC, white blood cell; eos, eosinophil; IOP, intraocular pressure; TB, tuberculosis.

A novel finding of this study was the spontaneous emergence of a worm from the eyelid. We strongly suspect that the worm emerged from the elder twin sister's left eyelid is a juvenile worm of *Paragonimus skrjabini*, based mainly on its emergence from the biopsied tunnel shortly after biopsy and its morphological appearance. Moreover, mNGS of the puncture sample from the elder twin sister's eyelid subcutaneous tissue confirmed the presence of *Paragonimus skrjabini*. Thus, the emerged worm is most likely to be a juvenile worm of *Paragonimus skrjabini*. We speculated that this might have been triggered by the subcutaneous puncture, which created a subcutaneous tunnel and stimulated the juvenile worm, facilitating its emergence. While ectopic migration is documented (21), direct visualization of a live worm emerging from ocular tissue is exceptional. Ultimately, the diagnosis was confirmed by mNGS and the presence of anti-*Paragonimus* antibody in the serum detected by ELISA. These are accurate diagnostic tools for *P. skrjabini* infection when the conventional methods such as stool examination are not sensitive. However, there are also limitations; although a worm emerged, histologic proof that it was a *Paragonimus* fluke worm was not established; there is still no concrete diagnostic algorithm for ectopic infection. Eosinophilia and thrombocytosis were detected in both patients; which is consistent with previous reports stating that eosinophilia and thrombocytosis were common in patients with thoracopulmonary paragonimiasis (22). Recent data suggest that pulmonary inflammation may induce local megakaryopoiesis, leading to thrombocytosis (23), which may have been the reason for simultaneous eosinophilia and thrombocytosis in this patient. Although these cases were successfully treated with anti-parasitic medication, the long-term prognosis for ectopic infection, particularly when it involves more than one system, remains unknown. Serial imaging studies and long-term follow-up are mandatory.

## Conclusion

In conclusion, this case demonstrates the diagnostic challenges and spectrum of illness due to *P. skrjabini* infection. It highlights the importance of integration of molecular diagnostics with clinical and epidemiological data in managing rare parasitic diseases. The clinical manifestations of paragonimiasis are diverse and pediatric paragonimiasis often presents as multicentric involvement. Symptoms are non-specific and thus are prone to misdiagnosis. Children have a higher chance of exposure to infection as they frequently play outdoors and have different eating habits in some regions including consumption of raw freshwater crustaceans (24). It is recommended that targeted health education programs be implemented in endemic areas to change eating habits and advocate against consumption of undercooked food which can help prevent such infections.

## Data Availability

The original contributions presented in the study are included in the article/Supplementary Material, further inquiries can be directed to the corresponding author.
